# A Comprehensive Method for Predicting Fatal Liver Failure of Patients With Liver Cancer Resection

**DOI:** 10.1097/MD.0000000000000784

**Published:** 2015-05-01

**Authors:** Jiangfa Li, Biao Lei, Xingju Nie, Linku Lin, Syed Abdul Tahir, Wuxiang Shi, Junfei Jin, Songqing He

**Affiliations:** From the Department of Hepatobiliary and Pancreatic Surgery (JL, LL, SAT, SH); Laboratory of Hepatobiliary and Pancreatic Surgery (JL, BL, JJ, SH), Affiliated Hospital of Guilin Medical University; Guangxi Key Laboratory of Molecular Medicine in Liver Injury and Repair (JJ, SH); School of Public Health (WS), Guilin Medical University, Guilin, Guangxi, People's Republic of China; and Department of Radiology and Radiological Science (XN), Center for Biomedical Imaging, Medical University of South Carolina, Charleston, South Carolina, USA.

## Abstract

There are many methods to assess liver function, but none of them has been verified as fully effective. The purpose of this study is to establish a comprehensive method evaluating perioperative liver reserve function (LRF) in patients with primary liver cancer (PLC).

In this study, 310 PLC patients who underwent liver resection were included. The cohort was divided into a training set (n = 235) and a validation set (n = 75). The factors affecting postoperative liver dysfunction (POLD) during preoperative, intraoperative, and postoperative periods were confirmed by logistic regression analysis. The equation for calculating the preoperative liver functional evaluation index (PLFEI) was established; the cutoff value of PLFEI determined through analysis by receiver-operating characteristic curve was used to predict postoperative liver function.

The data showed that body mass index, international normalized ratio, indocyanine green (ICG) retention rate at 15 minutes (ICGR15), ICG elimination rate, standard remnant liver volume (SRLV), operative bleeding volume (OBV), blood transfusion volume, and operative time were statistically different (all *P* < 0.05) between 2 groups of patients with and without POLD. The relationship among PLFEI, ICGR15, OBV, and SRLV is expressed as an equation of “PLFEI = 0.181 × ICGR15 + 0.001 × OBV − 0.008 × SRLV.” The cutoff value of PLFEI to predict POLD was −2.16 whose sensitivity and specificity were 90.3% and 73.5%, respectively. However, when predicting fatal liver failure (FLF), the cutoff value of PLFEI was switched to −1.97 whose sensitivity and specificity were 100% and 68.8%, respectively.

PLFEI will be a more comprehensive, sensitive, and accurate index assessing perioperative LRF in liver cancer patients who receive liver resection. And keeping PLFEI <−1.97 is a safety margin for preventing FLF in PLC patients who underwent liver resection.

## INTRODUCTION

Currently, surgical resection is a preferred treatment option for patients with liver cancer except for liver transplant.^1^ Because of postoperative liver failure, severe complications like shock and death take place in many patients after liver resection.^2–4^ Liver reserve function (LRF) after resection, risk factors leading liver dysfunction or failure, methods for preventing these risk factors, and postoperational liver regeneration or repair have become the focus of research. When pursuing the elimination of the target lesion, 1 of the most critical measurements for liver cancer is to ensure the safety of the implementation of treatment programs and obtain the best effects of the eventual rehabilitation in patients. In order to achieve the desirable outcome, it is essential to have not only excellent surgical technique but also a good system that can assess the status of LRF in patients about to receive liver resection.

Indocyanine green (ICG) clearance test is a sensitive and accurate method that assesses LRF quantitatively.^5–7^ In the process of liver resection, many factors affect the perioperative LRF. These factors mainly include liver resection volume, hypoxia injury caused by operative bleeding, ischemia reperfusion injury, and other liver damages caused by intraoperative blood transfusion, stretch, squeeze, and anesthetic drugs. Although a single ICG clearance test is an accurate, quantitative assessment for preoperative LRF, it cannot make a comprehensive evaluation for perioperative LRF. After taking into consideration the main preoperative, intraoperative, and postoperative factors, one can achieve a comprehensive assessment for perioperative LRF.

In previous studies, many researchers use preoperative methods to evaluate LRF, and some of them already consider postoperative influencing factors, such as standard remnant liver volume (SRLV). SRLV, which is an effective, simple indicator assessing LRF in liver cancer patients about to receive liver resection, is defined as remnant liver volume (RLV) divided by body surface area (BSA). It plays an important role guiding the prediction of LRF of postoperative patients and thus helps preventing postoperative liver failure.^8^ Varieties of studies indicate that intraoperative factors significantly affect postoperative liver function,^9–14^ especially intraoperative bleeding and operating time; however, none of these intraoperative factors is brought into assessing methods. It is well known that excessive bleeding might increase perioperative mortality or complications that affect long-term survival in patients.^9–11^ Therefore, evaluation can be more effective if intraoperative factors are taken into consideration when assessing perioperative LRF.

In this study, we combined multiple perioperative factors to evaluate postoperative liver function in PLC patients and established an equation calculating the preoperative liver functional evaluation index (PLFEI) that combines preoperative, intraoperative, and postoperative factors influencing liver function. We confirmed that PLFEI acts as a sensitive, comprehensive, objective, and effective indicator for evaluating perioperative liver function in liver cancer patients about to receive resection.

## METHODS

### Study Design

First, we analyzed the clinical data of 235 patients who underwent liver resection between September 2010 and April 2014 at the Affiliated Hospital of Guilin Medical University, Guilin, China. We identified the determinants and established a logistic regression model for evaluating perioperative liver function in patients with PLC. Second, we proposed a new indicator PLFEI calculated by a logistic regression equation; the cutoff value of PLFEI was determined by receiver-operating characteristic (ROC) curve analysis. Third, we evaluated the validity and reliability of PLFEI using an independent set including 75 patients who underwent liver resection at the same institutes from March 2014 to October 2014.

### Postoperative Liver Dysfunction Criteria

Postoperative liver dysfunction was defined as the total bilirubin levels ≥50 μmol/L and/or prothrombin time (PT) index <50% at fifth postoperative day.^15^ Patients were followed up for 1 month after liver resection. The postoperative patients were divided into 2 groups: Group A without liver dysfunction and Group B with liver dysfunction.

### Patients

The local ethics committee approved this study protocol; all patients included in this study signed an informed consent. Three hundred ten consecutive PLC patients who underwent liver resection were enrolled in the study at the Affiliated Hospital of Guilin Medical University.

The training set recruited 235 cases of patients. Two hundred six patients were males and 29 were females; the average age was 50.41 ± 11.01 years (ranging from 24 to 84 years). Postoperative pathologic examination confirmed 195 (82.98%) patients as having hepatocellular carcinoma and 40 patients (17.02%) as having cholangiocarcinoma. The average diameter of tumors in these patients was 8.5 ± 5.2 cm (ranging from 3.0 to 20.2 cm); 215 (91.49%) patients had a history of hepatitis B or their HBsAg was positive, and hepatitis C antibody in 10 cases (4.26%) was positive. Three of 10 patients without viral hepatitis were diagnosed as having nonalcoholic fatty liver disease (NAFLD).

Seventy-five patients were included in the validating set; 61 patients were males and 14 cases were females, and the average age was 46.3 ± 11.4 years (ranging from 28 to 81 years). Among them, postoperative pathologic examination determined 61 (81.33%) patients as having hepatocellular carcinoma and 14 (18.67%) cases as having cholangiocarcinoma. The average diameter of these tumors was 9.1 ± 4.2 cm (ranging from 4.0 to 18.5 cm). Sixty-six (88.0%) patients had a history of hepatitis B or their HBsAg was positive, and hepatitis C antibody in 3 cases (4%) was positive.

### ICG Test

All the patients in this study conducted an ICG clearance test at 1 to 3 days before operation. ICGR15 data was obtained from Pulse Dye Densito-Graph Analyzer (DDG-3300K; Nihon Kohden, Tokyo, Japan). After an optical sensor of the ICG clearance meter was attached to a patient's index finger, ICG (5 mg/mL, Dandong Medical and Pharmaceutical Co., Liaoning, China) was intravenously administered at a dose of 0.5 mg/kg body weight via central venous catheter that was immediately flushed with normal saline.

### INR and BMI

PT was determined at 1 to 3 days before operation and on the postoperative days 1, 3, 5, and 7 using PT test kit (Nanjing Jiancheng technology Co., Ltd, Nanjing, China) and automatic blood coagulation instrument (Sysmex, Japan) in accordance with the manufacturer's instructions. International normalized ratio (INR) = (patient PT/control PT)^ISI^. Body mass index (BMI, kg/m^2^) = body weight (kg)/body height (m)^2^. The patients were divided into 4 grades according to the BMI^16,17^: underweight (<18.5), normal (18.5–24.9), overweight (25.0–29.9), and obesity (>30.0 kg/m^2^).

### Liver Volume Measurement

All the patients received a computed tomography (CT) scan (64-slice spiral CT; General Electric Co., Fort Myers, FL) before surgery. A parallel 3-dimensional of the liver was reconstructed to evaluate total liver volume (TLV, mL) and tumor volume. Liver resection volume (LRV, mL) was measured by water displacement method during operation. The RLV (mL) = TLV − LRV. SRLV (mL/m^2^) = RLV/BSA (m^2^). BSA (m^2^) = body weight (kg)^0.425^ × body height (cm)^0.725^ × 0.007184.^18^

### Liver Cirrhosis and NAFLD

According to the preoperative Child–Pugh score classification, liver cirrhosis was graded into 3 groups^19^: mild cirrhosis (Child–Pugh A), moderate cirrhosis (Child–Pugh B), and severe cirrhosis (Child–Pugh C). NAFLD was diagnosed according to the postoperative pathological examination, and segmented into steatosis, nonalcoholic steatohepatitis, and cirrhosis.^16,20,21^ Patients should not be diagnosed as NAFLD whose liver fat lesions were caused by other common liver diseases, particularly hepatitis C, hepatitis B, and alcoholic liver disease.^16,20,21^

### Operative Time and Bleeding Volume

Operative time was defined as the interval between open and closure of skin. Operative bleeding volume (OBV, mL) was the amount of blood that the patient lost during operation, and was calculated from the start to the end of the operation. The volume of blood loss was the suction volume minus rinsing fluids, and adding the weight of blood absorbed by swabs (assuming that 1 mL = 1 g).^22,23^

### Surgical Methods

All patients were given intravenous anesthesia and the selection of surgery method was based on results of operative exploration, preoperative examination-related indicators (ICGR15, CT, Child–Pugh classification, etc.), and the size and location of the tumor.

The methods of liver resection for the training set (n = 235) were summarized as follows: nonanatomic liver resection or local tumor resection (n = 109), left hemihepatectomy (n = 42, one of them with partial resection of the caudate lobe), right hemihepatectomy (n = 37, two of them with partial resection of the caudate lobe), mesohepatectomy (n = 13), left lateral liver resection (n = 19), right posterior lobe resection (n = 13), and extensive liver resection (n = 2). Surgical specimens of all the patients were routinely sent for pathological investigation. Two hundred ten patients had cirrhosis. Twenty patients had left or right branch of portal vein thrombosis and no one had trunk thrombosis. Thirty patients had liver resection without portal clamping. Thirty-seven patients had selective hemihepatic portal clamping and 40 patients had intermittent Pringle maneuver. The protocol for total portal occlusion did not exceed 15 minutes (9.2 ± 2.6 minutes per time) and the time interval of 2 consecutive blocks was >5 minutes. Thirty-two patients submitted to a liver resection received no blood transfusion.

The methods of liver resection for the validation set (n = 75) were summarized as follows: nonanatomic liver resection or local tumor resection (n = 33), left hemihepatectomy (n = 12), right hemihepatectomy (n = 7), the caudate lobe resection (n = 6), mesohepatectomy (n = 7), left lateral liver resection (n = 8), and right posterior lobe resection (n = 2). Surgical specimens of all the patients were routinely sent to pathological investigation. Sixty-seven patients had cirrhosis. Ten patients had left or right branch of portal vein thrombosis and no one had trunk thrombosis. Thirty-six patients had liver resection without portal clamping. Eighteen patients had selective hemihepatic portal clamping and 10 patients had intermittent Pringle maneuver. Twenty patients received blood transfusion during liver resection.

### Statistical Analysis

The continuous variables were expressed as mean ± standard deviation, and the means comparison between the 2 groups were examined by *t* test. The rate comparison between the 2 groups was done by χ^2^ test. The model was conducted by logical regression analyses and the formula predicting the status of postoperative liver function was obtained from the logical regression model mentioned earlier.

Most of statistical analyses were completed by SPSS19.0 statistical software. The ROC curve analysis was done by the MedCalc analysis software 12.4.0.0 (Ostend, Belgium). *P* < 0.05 was considered statistically significance.

## RESULTS

### Characteristics of Postoperative Patients Surveyed

Based on the postoperative liver dysfunction criteria, 204 patients were included into Group A and 31 patients were included into Group B. The group B was further divided into 2 subgroups: subgroup I, with 27 patients, all of whom recovered from POLD, and subgroup II, with 4 patients, all of whom died of liver failure. BMI, INR, ICGK, ICGR15, SRLV, OBV, blood transfusion volume, and operative time were statistically different between the 2 groups (*P* < 0.05), as shown in Table 1. Liver resections in 20 patients (13 in Group A and 7 in Group B) were >60% of TLV. LRV was not significantly different between the 2 groups (*P* = 0.235). The average OBV in patients was 733.6 ± 772.8 mL (range 100–5500 mL). Liver dysfunction occurred in 17 of 175 patients with OBV <1000 mL, which was lower than those with OBV >1000 mL (14/60, *P* < 0.01). None of 225 (95.74%) patients with viral hepatitis were found with obvious fat lesions in the liver, and 3 of 10 patients without viral hepatitis were diagnosed as NAFLD. In the 3 patients, 1 occurred with POLD and 2 without POLD. NAFLD had no significant difference between Group A and Group B, as shown in Table 1. In patients without virus hepatitis, NAFLD had no significant difference between the groups with or without POLD, as shown in Table 2.

**TABLE 1:**
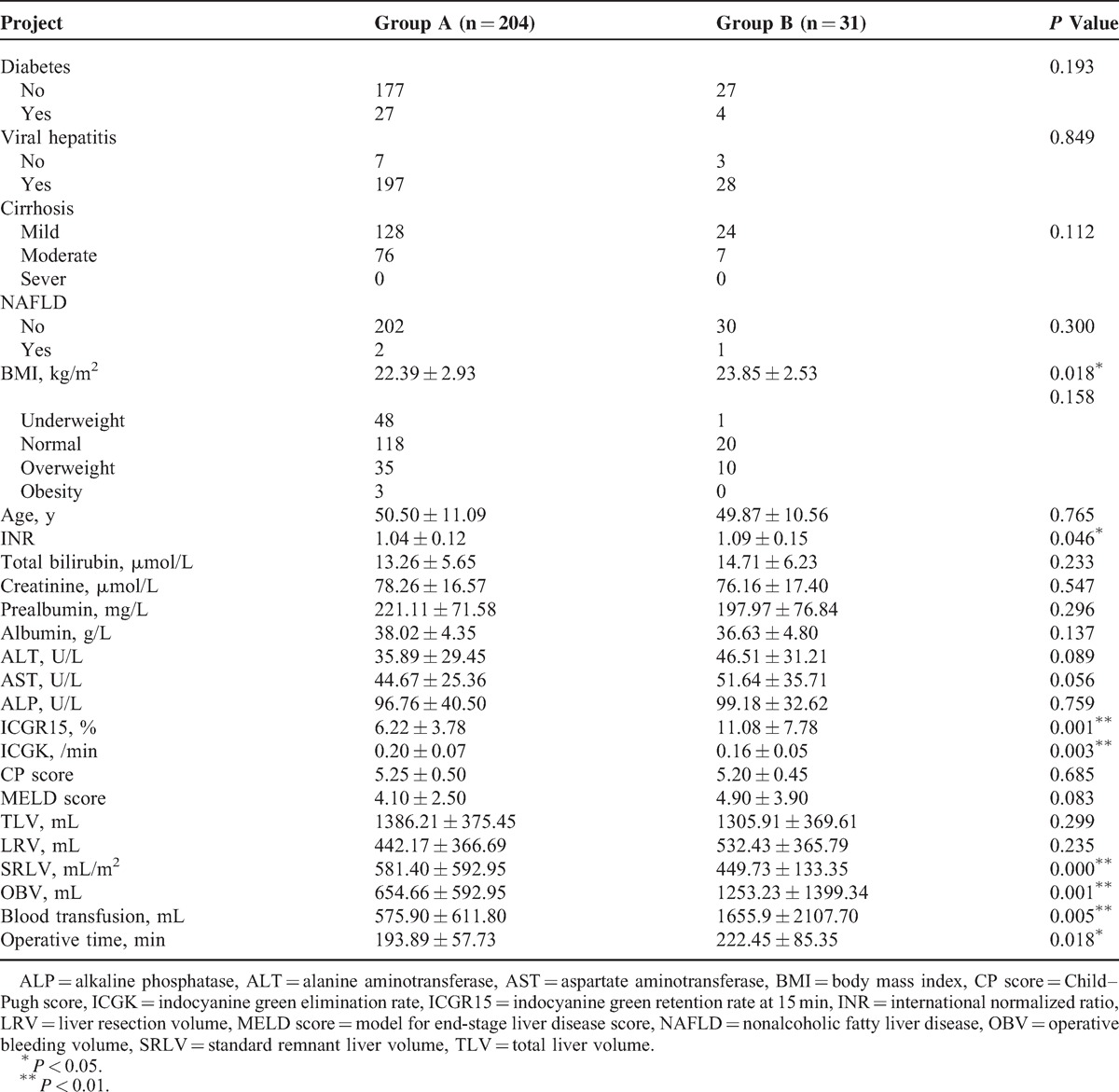
Comparison of Clinical Indexes Between Group A and Group B

**TABLE 2:**
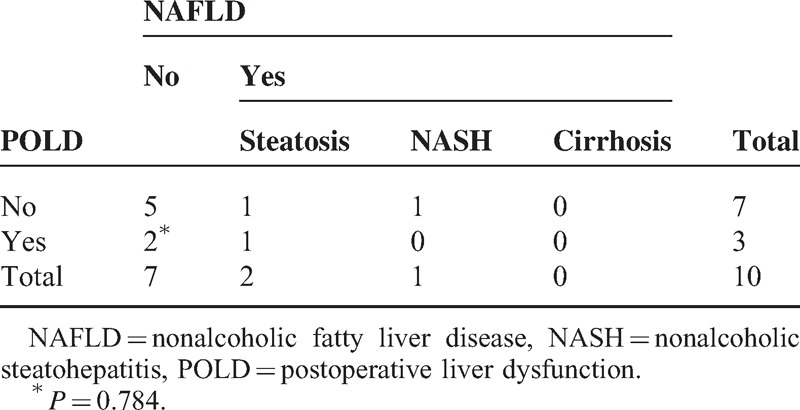
Comparison of NAFLD in Patients Without Virus Hepatitis

### Determinants of Postoperative Patients With Liver Dysfunction

The binary logical regression analysis was used to identify the relationship between the status of postoperative liver function and BMI, INR, ICGR15, ICGK, SRLV, OBV, blood transfusion volume, and operative time. The introduced variables were ICGR15, OBV, and SRLV and excluded variables were ICGK, INR, blood transfusion volume, operative time, and BMI by analysis using the forward stepwise method whose probability for entry and removal were 0.05 and 0.1, respectively. Therefore, the determinants of POLD were ICGR15, OBV, and SRLV as shown in Table 3. POLD was positively correlated with ICGR15 and OBV (all *P* < 0.05); however, it was negatively correlated with SRLV.

**TABLE 3:**
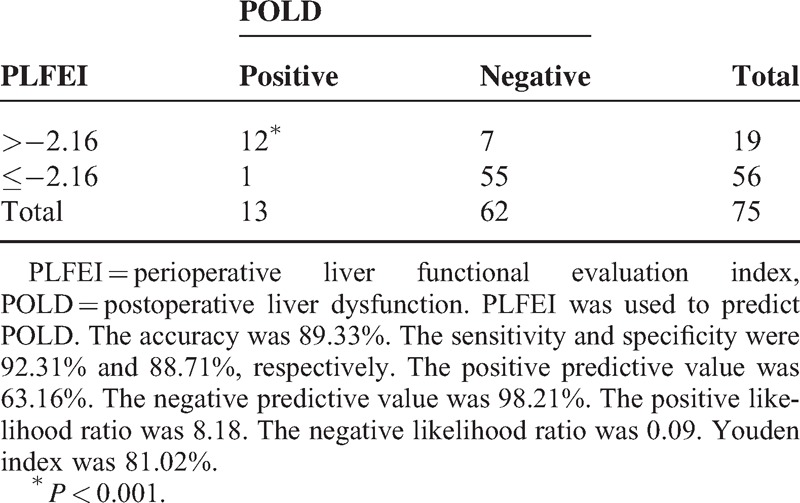
Covariates Included in the Logistic Regression Model (n = 235)

### PLFEI and the Cutoff

**FIGURE 1:**
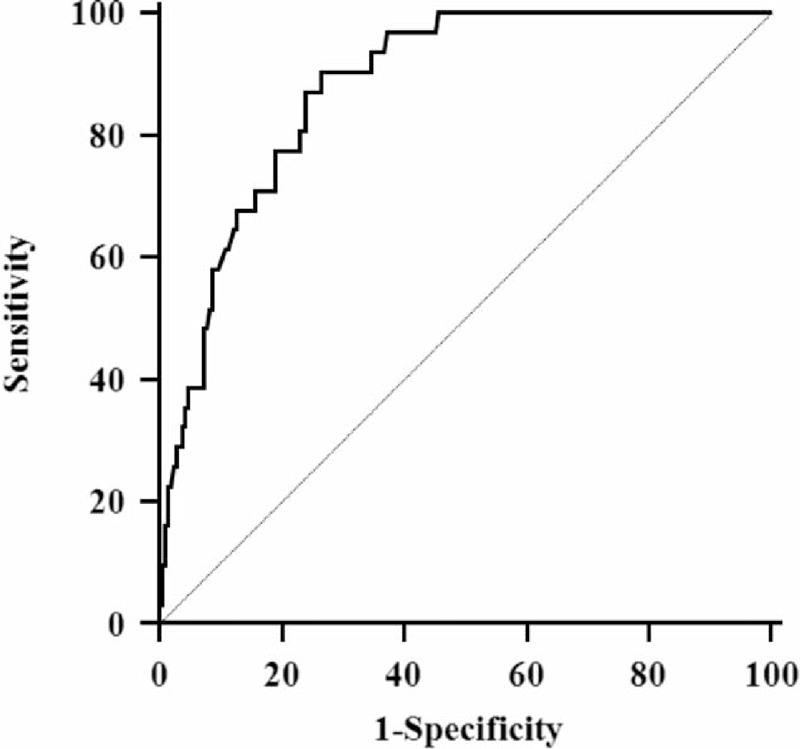
Sensitivity and specificity of a PLFEI value analyzed by ROC curve. The PLFEI value >−2.16 was used to predict POLD; its sensitivity and specificity were 90.30% and 73.5%, respectively. Area under the ROC curve was 0.879, standard error was 0.0261, and 95% confidence interval was from 0.830 to 0.918. PLFEI = preoperative liver functional evaluation index, POLD = postoperative liver dysfunction, ROC = receiver-operating characteristic.

**FIGURE 2:**
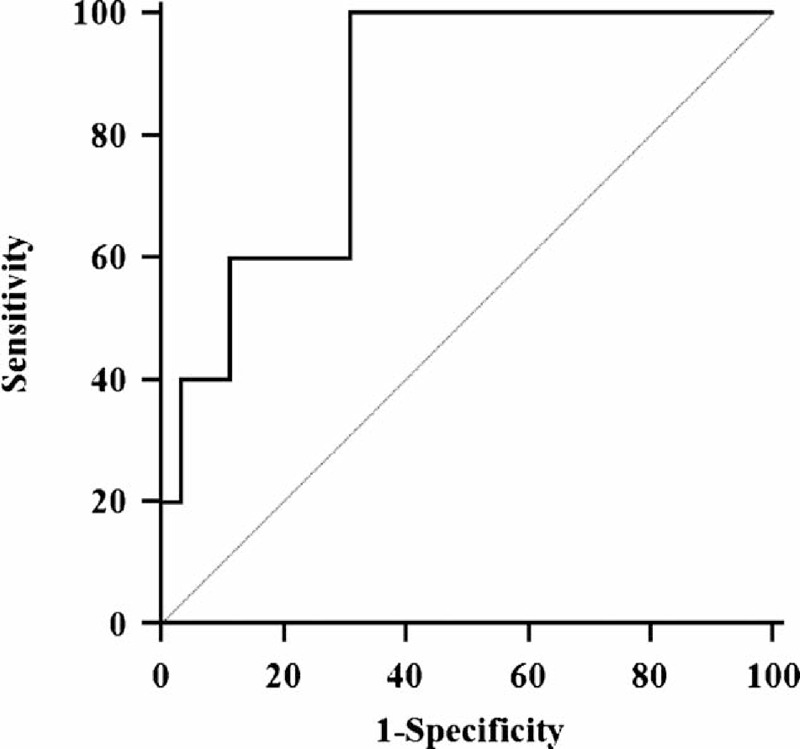
Sensitivity and specificity of a PLFEI value analyzed by ROC curve. The PLFEI value >−1.97 was used to predict FLF; its sensitivity and specificity were 100% and 68.8%, respectively. Area under the ROC curve was 0.885, standard error was 0.0709, and 95% confidence interval was from 0.837 to 0.923. FLF = fatal liver failure, PLFEI = preoperative liver functional evaluation index, ROC = receiver-operating characteristic.

**FIGURE 3:**
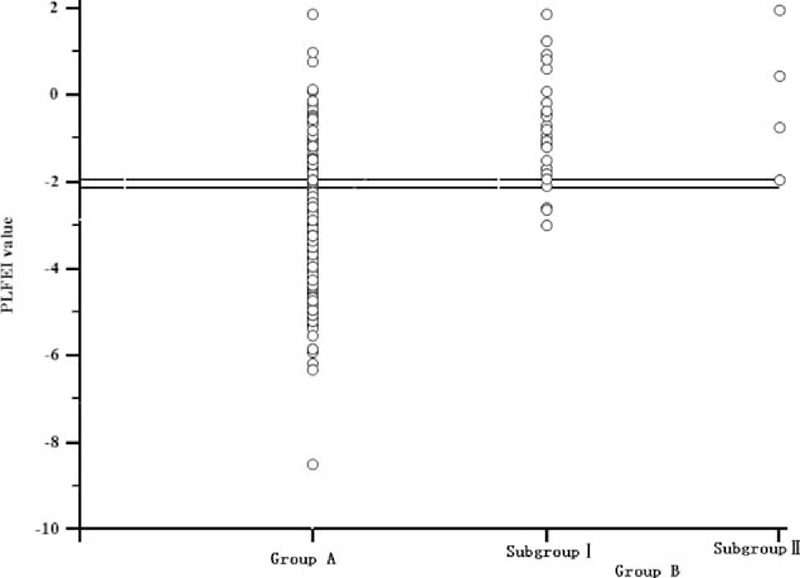
Majority of patients in Group A have a PLFEI value <−2.16, and the majority of patients in Group B have a PLFEI value >−2.16, and 4 patients who died of FLF have a PLFEI value >−1.97. FLF = fatal liver failure, PLFEI = preoperative liver functional evaluation index.

The logistic regression equation was established, namely, PLFEI = 0.181 × ICGR15 + 0.001 × OBV − 0.008 × SRLV. The PLFEI cutoff predicting POLD was −2.16 determined by ROC analysis from the training set; the sensitivity and specificity were 90.3% and 73.5%, respectively (Figure 1). The cutoff value of PLFEI to predict fatal liver failure (FLF) was −1.97 whose sensitivity and specificity were 100% and 68.8%, respectively (Figure 2). Postoperative FLF happened in all patients whose PLFEI was >−1.97 (Figure 3).

### Validity and Reliability of PLFEI

The sensitivity and the negative predictive value were >90% and the accuracy and the specificity were nearly 90% among the validating samples for predicting POLD (Table 4).

**TABLE 4:**
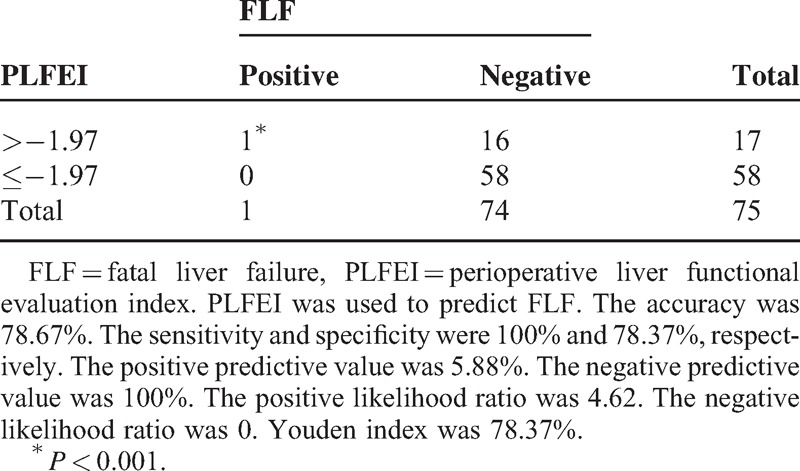
Evaluation of PLFEI in Predicting POLD

The sensitivity and the negative predictive value were 100% and the accuracy and the specificity were 80% among the validating samples for predicting FLF (Table 5).

**TABLE 5:**
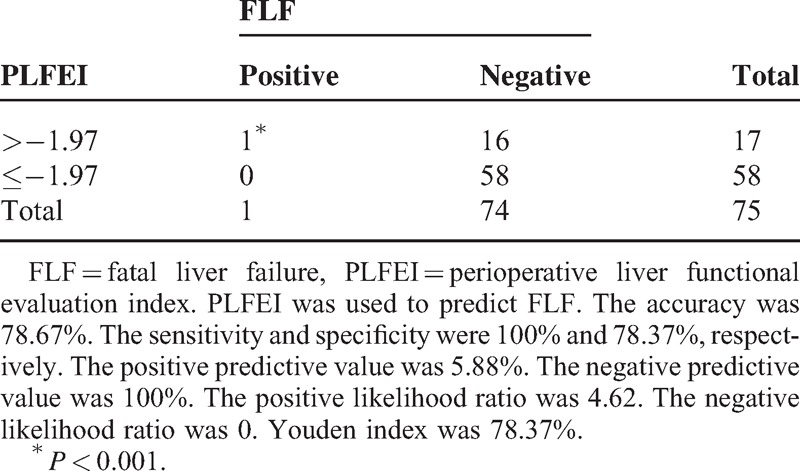
Evaluation of PLFEI in Predicting FLF

## DISCUSSION

### Main Influencing Factors for POLD

Many perioperative factors existing in preoperative, intraoperative, and postoperative phases are involved in POLD. First, some factors in preoperative phase affected postoperative liver function of patients with liver resection. Our data showed that preoperative ICGR15 among patients without POLD was lower than with POLD, which is similar to the result described by Okabe et al.^13^ Preoperative ICG clearance tests like ICGR15 and ICGK are clinically used to assess LRF, and many reports confirmed that they are safe, sensitive, and accurate quantitative methods evaluating LRF before liver resection; avoiding “high ICGR15” can prevent postoperative patients from liver dysfunction.^5,24^ We also found that preoperative INR among patients without POLD was less than with POLD, which resembles to the previous reports.^3,25^ PT was used as an independent prognostic factor in patients after liver resection when PT activity is >80%.^25^ Preoperative PT is >14 seconds; the death risk in postoperative patients will increase.^3^ Second, intraoperative factors had some effects on POLD. OBV in the group without POLD was less than with POLD. Bleeding in liver resection process is difficult to avoid. Excessive operative bleeding not only causes liver tissue hypoperfusion, which subsequently leads to liver tissue hypoxia/ischemia injury, but also affects postoperative hypoalbuminemia and aggravates the burden of liver. A large number of studies have shown that excessive operative bleeding significantly increases postoperative morbidity and mortality, especially in the patients with cirrhosis.^9–12^ Operating time in the group without POLD was shorter than with POLD. The duration of surgery is directly proportional to the postoperative recovery of patients. The prolonged surgery affects the liver function recovery and causes higher morbidity in patients after liver resection.^13,14^ Finally, some postoperative factors influenced POLD. The SRLV in the group without POLD was larger than with POLD. Liver volume is closely related to LRF.^26^ When RLV is below a certain threshold, the patient is prone to liver dysfunction, even FLF, and high mortality rate.^13^ SRLV is used as an even more individualized approach to evaluate residual LRF of patients.

NAFLD, abnormal BMI, and diabetes are considered as risk factors of complications after liver resection.^27–31^ In western countries, NAFLD is a prevalent problem.^32^ But in China, >90% of liver cancer patients infected with hepatitis B or hepatitis C,^33^ and most of them had liver cirrhosis. In agreement with this report, there were >90% patients with viral hepatitis and only 3 patients with NAFLD were observed in our study, therefore, viral hepatitis was the major whereas NAFLD was the minor factor leading to cirrhosis that affected LRF. BMI had a significant difference between patients without and with POLD, *P* = 0.018. But, there was no significant difference between patients without and with POLD in BMI by grade, *P* = 0.158, and no significant difference among four subgroups of BMI by multiple comparisons, similar to some study.^34–36^ This study showed that diabetes had no significant differences between patients without and with liver dysfunction, similar to the literature.^37^

In conclusion, we found that 3 preoperative determinants such as higher BMI, higher ICGR15, and larger INR affected POLD in patients with liver resection; 3 risk factors during operation like massive OBV, too much blood transfusion, and longer operation time played key roles in postoperative liver function insufficiency; 1 postoperative factor like undersized SRLV caused by excessive excision of liver tissue also influenced postoperative liver function.

### Validity and Reliability of PLFEI in Predicting the Status of Postoperative Liver Function for Patients With Liver Cancer Resection

We proposed the PLFEI and tried to include intraoperative influencing factor into the postoperative liver function evaluation system. When PLFEI is >−2.16, the sensitivity was 90.3% and the specificity was 73.5% for evaluating POLD. When PLFEI is >−1.97, the sensitivity was 100% and the specificity was 68.8% for evaluating FLF. Many evaluation methods of liver function only rely on the detection of preoperative LRF, and some methods pay close attention to the postoperative RLV. Although a variety of studies suggested that some intraoperative factors significantly affect the postoperative liver function,^9–14^ there was not a single evaluation method that took intraoperative factors into considerations. PLFEI includes intraoperative influencing factor and it is more comprehensive and objective evaluation method for perioperative liver function and for prediction of postoperative liver function. It is known that operation time and OBV may vary owing to different surgeons, the patient's postoperative outcome is likely to be different. Therefore, when preoperative liver function evaluation method was used to predict the maximum excision volume of liver, intraoperative influencing factors should not be ignored.

### Clinical Application of the PLFEI

PLFEI might be evaluated even before surgery. Every patient could have a preoperative ICG test to obtain the ICGR15 value, TLV and LRV could be estimated through a CT scan before surgery, and then SRLV could be figured out. Rough OBV could be estimated according to imaging data and doctors’ experience. If there is a less estimated OBV and a higher estimated PLFEI value, this patient should be dealt with more carefully. Especially when the estimated PLFEI is >−1.97, the surgery should be carefully designed to ensure the safety of patients, even giving up the surgery. During the surgery, bleeding should be reduced as far as possible, and some effective hemostatic measures and new surgical devices should be taken.^22^

We can calculate and find out the PLFEI immediately after the surgery. Before surgery, the preoperative ICGR15 value could be determined, the TLV can be estimated through a CT scan, the LRV can be obtained during surgery, OBV can be obtained immediately after the surgery, and then the PLFEI will be figured out. Postoperative treatment, especially protecting liver therapy, should be enhanced in the patient with a higher PLFEI value, in order to reduce POLD.

Therefore, we conclude that PLFEI will be a more comprehensive, sensitive, and accurate index assessing perioperative LRF in liver cancer patients who receive liver resection, and keeping PLFEI <−1.97 is a safety margin for preventing FLF in PLC patients after liver resection.

### Disadvantages of This Study

With all the mentioned advantages of PLFEI in mind, however, some disadvantages need to be considered. First, the number of cases is relatively small. Second, there are only 3 patients with NAFLD involved in this study and no patient with severe cirrhosis. Finally, OBV is difficult to be estimated accurately before surgery. Therefore, the related comprehensive evaluation methods need further study.

## CONCLUSIONS

PLFEI will be a more comprehensive, sensitive, and accurate index assessing perioperative LRF in liver cancer patients who receive liver resection. Also keeping PLFEI <−1.97 is a safety margin for preventing FLF in PLC patients after liver resection.
