# A Comprehensive Method for Predicting Fatal Liver Failure of Patients With Liver Cancer Resection: Erratum

**DOI:** 10.1097/01.md.0000466603.09468.0e

**Published:** 2015-06-05

In the article “A Comprehensive Method for Predicting Fatal Liver Failure of Patients With Liver Cancer Resection”,^1^ which appeared in Volume 94, Issue 17 of *Medicine*, a manufacturer location was incorrectly listed. Reference 18 was also incomplete. The article has since been corrected online.
